# Observations on the Utility of Amputating the Extremities of the Bones in Cases of Caries of the Joints

**Published:** 1803-12-01

**Authors:** M. Moreau


*Observations on the Utility of amputating the Extremities of the Bones in Cases of Caries of the Joints; by* M. MOREAU, M. D. *Communicated by our Correspondent at Paris.*

THE Author details a number of Cases where this mode of treatment superseded the necessity of amputating the entire extremity, and even without any considerable defect

---

fect in the portions of the member. Although we find similar cases in the Memoirs of the Academy of Surgery, and in the works of Mr. White of Manchester, Mr. Parke, &c. yet we conceive it will be interesting to offer some extracts from the work of M. Moreau, whose practice, as well as that of his father's, tends to generalize very much the utility of this method. The first observation is that of a young man, nineteen years of age, with an enlarged elbow joint, which came on after a swelling of the axillary glands; abscesses had formed in the neighbourhood of the joint, communicating with its cavity, and opening externally. The operation is thus described: The patient was laid on his belly on a table, the posterior part of the arm turned upwards, and lying close to the edge of the table. With a scalpel he cut down, in the direction of the inner condyle, to the articulation; a similar incision was made on the opposite side, and the two were united by one across dividing the skin and tendon of the triceps, immediately above the olecranon. Having exposed the parts, he found the humerus bare and covered with asperities, the articulation full of pus and fungosities, &c. After having detached the flesh from the anterior part of the bone above the condyles, he placed the handle of the scalpel between the bone and the fleshy parts, and he sawed the bone directly over it, and in this way he detached the bone from the surrounding parts; on removing which, he discovered that the diseases of the bone extended farther, and which obliged him to cut some lines more of the bone. His attention was next turned to the fore arm; an incision was made from the external condyle along the edge of the radius; the head of this bone was separated from the surrounding parts, as well as its union with the ulna. A band of linen was interposed to protect the soft parts, and the bone was cut above the insertion of the biceps muscle, which it was of importance to preserve. A new incision, corresponding to that of the internal condyle of the humerus, laid the radius bare, and which, with the incisions already described, formed a quadrilateral flap, adhering at one side to the flesh, on the posterior part of the fore arm. This flap was equally detached from the diseased part of the bone, which he sawed at about an inch and a half from the olecranon. Notwithstanding the length of time the operation required, and the extent of the incisions, the wound was nearly healed the seventh day. Two years have elapsed since the operation, and the patient had so far recovered the use of this arm as to be able to thresh corn.

corn. The extremity is shorter by three inches, the size of the limb somewhat diminished, but the person is daily acquiring the natural use of the part. Two similar operations, attended with equal success, were performed by the author's father.

The fourth observation of the author relates to an operation in the knee-joint; the state of the articulation and adjoining bones was nearly similar to the one described. The operation was performed by making an incision above the condyles, on each side of the thigh, between the vasti and flexor muscles; these incisions were united by a transverse one down to the bone, passing above the patella. Having detached the flap from the bone, on bending the knee the state of the articulation was perceived, and the bone was sawed above the part that appeared diseased. In order to remove the superior part of the tibia, an incision was carried along the sharp edge of the tibia, and one of equal extent was made on the head of the fibula on its external side. The consequence was, one flap composed of the flesh situated in the interossial space externally, and a second triangular one, formed by the skin covering the internal surface of the tibia; the head of the fibula was first laid bare and sawed, after that the condyles of the tibia were separated about ten lines in length. Every thing succeeded after the operation, till the patient was seized with a dysentery, prevalent in the hospital, which carried him off in fifteen days.

The operations before described have been already performed by other surgeons, before M. Moreau, but the following, we believe, to have been attempted by him only. His fifth operation was on the ancle-joint; we omit the the details of this operation, since the principle directing the former operations apply here. It is useful to remark, that in these kind of cases, great embarrassment arises from the difficulty of exposing the bone sufficiently for the saw, considering the necessity for preserving the tendons, vessels, &c. surrounding this articulation. The difficulties do not exist in the articulations of the bones of the tarsus, the spongy structure of those bones is a reason why caries is seldom confined to the joints, and in which case the total removal of the bone became an easier operation. This is not the case with the os calcis; here it is necessary to preserve the attachment of the muscles, and work out the diseased part of the bone, as the author did in one instance; who, in the case where it is necessary to destroy this attachment, recommends taking off the limb. With respect respect to the bones of the metatarsus, notwithstanding that they may be carious, either entirely or partially, he recommends their extirpation when the disease extends through a great part of the bone; for in this case, the bones of the corresponding toes lose their support. If, on the other hand, the caries is confined to the base, the section of the diseased part only should be attempted. He observes, that he never experienced any troublesome bleeding from the arteria plantaris, and never found it necessary to tie it.

The author performed the operation once, and with success, on the wrist joint; he has not detailed the operation, but we presume the principles to be the same as those laid down in the operation at the ankle. In the case in which he performed it, the person recovered almost entirely the use of the joint.

The last operation which the author mentions, is one on the shoulder joint. An incision was made on the posterior part of the articulation, beginning at the apophysis acromion, extending downwards two inches; it was parallel to another incision, distant from the former about four inches, and which had been previously made, in order to give issue to a collection of matter that had been formed. A transverse incision was made superiorly, at six lines under the superior insertion of the deltoid; this flap was turned down, in order to expose the articulation. At each extremity of the transverse incision a new one was made, the anterior directed towards the humoral extremity of the clavicle, and the posterior towards the spine of the scapula; this formed a second flap, and by means of which he could discover the entire extent of the caries; the head of the bone, disengaged from its cavity, was sawed; the parts united in the usual manner, soon healed, and the shoulder had the appearance as if dislocated; the superior part of the humerus took a position on the ribs at the edge of the scapula anteriorly. The motions of the arm remained very perfect, except the power of elevation, which was very limited. In all the cases related, the lips of the wounds were united by sutures; in one case, where he could not employ them, he found that the parts did not unite so quickly.

SCARPA'S

